# Three Novel COVID-19 Pneumonia Cases Successfully Treated With Lopinavir/Ritonavir

**DOI:** 10.3389/fmed.2020.00241

**Published:** 2020-05-19

**Authors:** Tatsuhiko Wada, Kosuke Shimode, Takayuki Hoshiyama, Yoko Takayama, Kunihiro Yamaoka

**Affiliations:** ^1^Department of Rheumatology and Infectious Diseases, Kitasato University School of Medicine, Sagamihara, Japan; ^2^Department of Infection Control and Prevention, Kitasato University Hospital, Sagamihara, Japan

**Keywords:** COVID-19, Lopinavir, Ritonavir, elderly patient, hyponatremia, cruise ship

## Abstract

Following the first case of Coronavirus Disease 2019 (COVID-19), caused by Severe Acute Respiratory Syndrome Coronavirus-2 (SARS-Cov-2), in Wuhan, China, in December 2019, it has spread worldwide. An outbreak in Japan occurred on a cruise ship, and this was followed by community-acquired COVID-19. Herein, we report three cases of COVID-19 that presented pneumonia following admission to Kitasato University Hospital. Patients were admitted based on the positive result of real-time reverse transcriptase–polymerase chain reaction (RT-PCR) tests for COVID-19 nucleic acid. All patients were diagnosed as suffering from non-severe COVID-19 pneumonia and were successfully treated with Lopinavir/Ritonavir (LPV/r). LPV/r could be an option for treating non-severe COVID-19 pneumonia in general and even in elderly patients.

## Introduction

Coronavirus Disease 2019 (COVID-19), caused by Severe Acute Respiratory Syndrome Coronavirus-2 (SARS-CoV-2), has spread worldwide, resulting in over 597,000 cases, and more than 27,000 deaths as of March 28, 2020. COVID-19 is characterized by fever, cough, and dyspnea, often followed by pneumonia (1). About 80% of cases are classified as mild, the other 20% are severe or critical (2). A recent study using multivariate analysis to identify the risk factors of COVID-19 pneumonia found that being elderly, male sex, and the presence of hypertension were independently associated with severe disease at admission, irrespective of adjustment of time to admission (3). In another report of 72,314 Cases from China, although 3% of confirmed cases were in those aged 80 years or older, the case-fatality rate was 14.8% in patients within that age bracket (2).

A COVID-19 outbreak in Japan occurred on a cruise ship. There were over 3,700 people aboard, and 634 passengers and/or crew tested positive for the coronavirus (4). Elderly people with background medical conditions disembarked for observation.

As there is no known effective treatment for COVID-19, the main treatment is supportive care. Attempts to use existing antiviral agents are believed to have been effective to a certain degree. Lopinavir is a protease inhibitor with activity against human immunodeficiency virus and has been formulated with ritonavir, which is a cytochrome P450 3A4 enzyme inhibitor, resulting in stabilization of Lopinavir concentration. The typical dose for HIV treatment is Lopinavir 400 mg/ritonavir 100 mg twice daily. During the outbreak of severe acute respiratory syndrome (SARS) in 2003, LPV/r was reported to inhibit the *in vitro* activity of the SARS coronavirus (5, 6). Based on this information, three patients with COVID-19 pneumonia were treated with LPV/r, following signed consent by all patients for treatment and compassionate use approval.

Herein, we report three cases of COVID-19 that presented pneumonia following admission to Kitasato University Hospital. Patients were admitted based on the positive result of real-time reverse transcriptase–polymerase chain reaction (RT-PCR) tests for COVID-19 nucleic acid. All patients were diagnosed as suffering from non-severe COVID-19 pneumonia and were successfully treated with Lopinavir/Ritonavir (LPV/r).

## Case Presentation

### Patient 1

A 60-year-old man with hyperlipidemia and with no history of hypertension, diabetes, or heart disease boarded the cruise ship 16 days before it went into quarantine. He noticed fever (day 0), RT-PCR positive was observed on day 4, and he was admitted to our hospital on day 7. His family members that had been in close contact with him tested negative. On admission, he had a fever and cough but had no difficulty in breathing. The physical examination showed no abnormalities. The results of blood examination were as follows: white blood cell (WBC) count 6,900/μL, lymphocyte count 1,056/μL, sodium level 131 mmol/L, C-reactive protein (CRP) 4.45 mg/dL.

Following continuous fever for 7 days, on day 13, oxygen saturation suddenly decreased to 88% at room air without any obvious symptoms such as dyspnea. We started oxygen inhalation, which had to be increased. Chest X-ray demonstrated infiltrative shadows at the bilateral lower lung, and chest computed tomographic (CT) scan revealed bilateral ground-glass opacity (GGO) and a crazy-paving appearance, and we diagnosed him with COVID-19 pneumonia. Blood examination showed a slight elevation of WBC count, with decreased absolute lymphocyte counts, hyponatremia, hiper-ferritinemia, and a high CRP level: WBC 7,900/μL, lymphocyte 711/mL, sodium level 132 mmol/L, Ferritin level 866 ng/mL, CRP 12.21 mg/dL. Due to the abrupt oxygen requirement, which had to be increased, and the results of the CT scan, Lopinavir/Ritonavir was started on day 13. Simvastatin 50 mg once daily for hyperlipidemia was switched to pravastatin due to contraindication of simvastatin during treatment with LPV/r. The body temperature and oxygen requirement decreased and lymphopenia normalized within 2 days, followed by improvements in other measures. Chest-CT images showed that subpleural curvilinear shadow, ground-glass pattern, and consolidation were improved on day 16. He was discharged from the hospital after testing RT-PCR negative twice.

### Patient 2

An 88-year-old Japanese male with a history of prostate cancer and hypertension boarded the cruise ship with his wife 16 days before the ship was quarantined. He recognized fever (day 0) and underwent RT-PCR testing on day 4, with a positive result, and was admitted on day 6. On admission, he suffered from fever and fatigue without cough or breathing difficulty. Blood results were as follows: WBC 3,100/μL, lymphocyte 704/mL, AST 39 IU/L, ALT 18 IU/L, sodium 127 mmol/L, and CRP 1.52 mg/dL. Fever and fatigue persisted, and other symptoms such as cough and diarrhea were developed on day 11. Despite our efforts to correct sodium abnormality, hyponatremia continued, ranging from 125 to 127 mmol/L, with a mild oxygen requirement (1–2 L/min). Chest X-ray revealed a new appearance of infiltrative shadow in the bilateral lung, and the CT scan image showed GGO in the outer area with or without consolidation. The diagnosis of COVID-19 pneumonia was made, and LPV/r was initiated after achieving consent. He had continued to take telmisartan 40 mg and trichlormethiazide 1 mg for hypertension, tamsulosin hydrochloride 0.2 mg for dysuria, and bicalutamide, an androgen receptor antagonist, 80 mg for prostate cancer. Symptoms gradually decreased in severity, with improvements in hyponatremia and lymphocytopenia. Although diarrhea and appetite loss due to LPV/r were seen, he was able to continue treatment. On day 25, he tested RT-PCR-negative for two samples and was discharged.

### Patient 3

A 44-year-old female without past medical history who is healthcare personnel and had close contact with COVID-19 pneumonia patients recognized fever without any other symptoms (day 0). An RT-PCR test was positive on day 2, and she was admitted to our hospital on day 3. On admission, she was symptom-free without any abnormality on physical examination. Her laboratory test results were as follow: WBC 3,600/μL, lymphocyte 1,224/mL, sodium level 136 mmol/L, Ferritin 20 ng/mL, and CRP 0.06 mg/dL. She developed a fever and dry cough on day 5 but had no severe respiratory symptoms such as difficulty in breathing, chest pain, or productive sputum. However, infiltrative shadows were observed in the right upper and left lower area on chest X-ray on day 6, and CT scan showed consolidations in the same areas on day 7. After obtaining consent, we started LPV/r on day 8, but she suffered from gastrointestinal adverse events and discontinued LPV/r on day 11. She had no concurrent medication. Improvement of pneumonia was observed on day 15, and she was discharged after two consecutive negative RT-PCR results. Interestingly, following the improvement of pneumonia without any symptoms or abnormality in blood examinations, RT-PCR remained positive during her follow-up RT-PCR testing.

**Figure 1:**
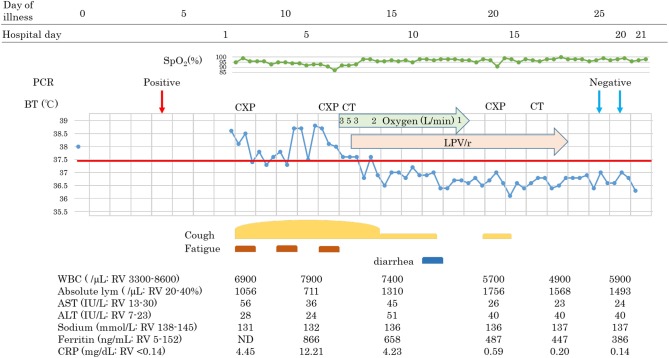
Clinical course of patient 1.

**Figure 2:**
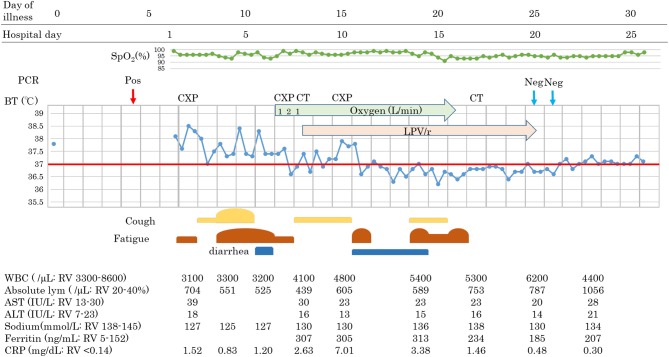
Clinical course of patient 2.

**Figure 3:**
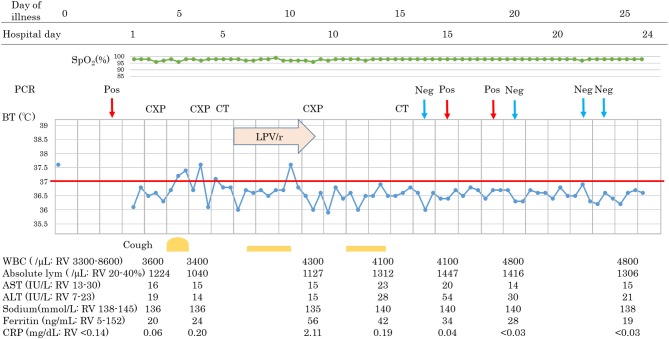
Clinical course of patient 3. BT, body temperature; CT, computed tomographic scan; CXP, chest X-ray photograph; Neg, negative PCR test; PCR, polymerase chain reaction test for COVID-19 nucleic acid; Pos, positive PCR test; RV, reference value.

**Figure 4:**
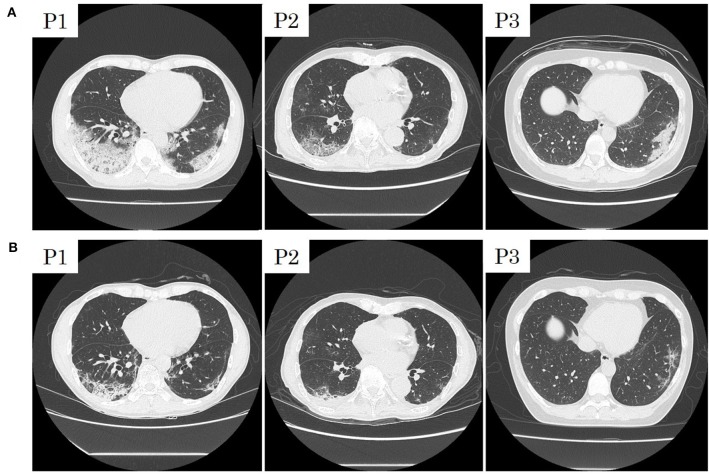
Initial computed tomographic images of patients 1 (P1), 2 (P2), and 3 (P3) with COVID-19 pneumonia **(A)** and those after treatment with Lopinavir/Ritonavir **(B)**.

Patients 1 and 2 developed a fever over 38 C and required oxygen inhalation, but neither were observed throughout the clinical course in Patient 3 (Figures 1–3). Pneumonia developed 8, 12, and 7 days after the onset of illness in Patients 1, 2, and 3, respectively (Figure 4; P1A, P2A, and P3A). After LPV/r was initiated, body temperature decreased, with improvement of cough, lymphopenia, hyponatremia, hyper-ferritinemia, and infiltrative shadows in Patients 1 and 2 (P1B, P2B). This effect was rapid in Patient 1 and gradual in Patient 2. No abnormal laboratory test results except for increased CRP were observed in Patient 3. The duration of treatment with LPV/r was 10, 12, and 3 days for patients 1, 2, and 3, respectively.

## Discussion

COVID-19 is spreading worldwide, although we are taking infection control measures such as frontline measures, isolation, and quarantine. One of the reasons why SARS-CoV-2 is rapidly transmitted between humans is that this virus has a 2-week incubation period before the onset of COVID-19. Moreover, there is no specific treatment or prophylaxis for COVID-19, such as antiviral agents and vaccines. Depending on poor information regarding COVID-19, especially on prognosis, we treated three patients with LPV/r.

Compared to the two elderly male patients, the young female patient presented very mild symptoms with no abnormal laboratory tests; low-grade fever and cough only. This is in line with the observation from China indicating milder symptoms in younger patients. Lymphocytopenia was observed in our elderly male patients, as reported previously (1, 7, 8). However, our cases are marked by the appearance of hyponatremia and hyper-ferritinemia, indicating a possible difference in laboratory tests results depending on age and sex. More importantly, an 88-year-old male patient was successfully treated with LPV/r for the first time. Since the median age of COVID-19 developing ARDS was 61, elderly patients are at high risk of severe respiratory dysfunction.

A randomized clinical trial of COVID-19 pneumonia patients treated with LPV/r conducted in China found no difference in time to clinical improvement and mortality at 28 days (9). However, our patients were heterogeneous concerning the duration and severity of illness at admission, and questions remain about whether earlier LPV/r treatment could have been effective for non-severe elderly COVID-19 pneumonia patients. Therefore, LPV/r could be an option for treating COVID-19 pneumonia in general and even in elderly patients.

We recognize the limitations of this case report. Only three patients were presented, and SARS-CoV-2 viral load and the blood concentration of LPV/r were not demonstrated, making it difficult to conclude whether LPV/r was effective on viral load. Cytokine level evaluation during the course of disease is another parameter that could provide a better understanding of the drug activity. The relationship between clinical course and serum cytokine level in COVID-19 patients requires future research.

## Data Availability Statement

All datasets presented in this study are included in the article/supplementary material.

## Ethics Statement

Ethical review and approval was not required for the study on human participants in accordance with the local legislation and institutional requirements. The patients/participants provided their written informed consent to participate in this study. Written informed consent was obtained from the individual(s) for the publication of any potentially identifiable images or data included in this article.

## Author Contributions

TW and KS wrote the manuscript with support from TH, YT, and KY. All authors contributed to manuscript revision, read and approved the submitted version.

## Conflict of Interest

The authors declare that the research was conducted in the absence of any commercial or financial relationships that could be construed as a potential conflict of interest.
